# The Relationship of Artificial Intelligence Opportunity Perception and Employee Workplace Well-Being: A Moderated Mediation Model

**DOI:** 10.3390/ijerph20031974

**Published:** 2023-01-20

**Authors:** Guanglu Xu, Ming Xue, Jidi Zhao

**Affiliations:** 1 School of Business, Nanjing University of Information Science & Technology, Nanjing 210044, China; 2 School of Business Administration, Shanghai Lixin University of Accounting and Finance, Shanghai 201620, China; 3 School of Public Administration, College of Economics and Management, East China Normal University, Shanghai 200062, China

**Keywords:** artificial intelligence opportunity perception, informal learning in the workplace, employee workplace well-being, unemployment risk perception

## Abstract

Several previous studies have revealed a positive relationship between artificial intelligence (AI) technology development and employees’ employment, income, and job performance. If individuals can seize the opportunity to master the knowledge and skills relevant to the implementation of AI, they could make career progress and improve their workplace well-being (WWB). Based on the transactional theory of stress and resource conservation theory, we constructed a moderated mediation model to explore the relationship between AI opportunity perception and employees’ WWB and examine the mediating factor of informal learning in the workplace (ILW), as well as the moderating factor of unemployment risk perception (URP). Through a survey of 268 employees, our results showed the following: (1) AI opportunity perception was significantly positively correlated with employees’ WWB; (2) ILW played a mediating role in the positive relationship between AI opportunity perception and employees’ WWB; and (3) URP negatively moderated the mediating relationship of ILW between AI opportunity perception and employees’ WWB. Our research results have a guiding significance for enterprises seeking to promote WWB during AI application.

## 1. Introduction

In 1956, the concept of “artificial intelligence” (AI) was first proposed at the Dartmouth Conference. However, due to the limitations of computer data processing, AI did not rapidly develop. Since the 1990s, improvements in computer pattern recognition and prediction abilities have correlated with rapid developments in AI [1]. The International Organization for Standardization defined an AI system as an engineered system that generates outputs, such as content, forecasts, recommendations, or decisions for a given set of human-defined objectives [2]. As a permeable technology, AI can be integrated with various industries in the economy and potentially change the original production and operation methods, which will also have a considerable impact on employment [3]. Compared with previous scientific and technological revolutions, the impact of AI on employment is all-directional and extremely intense, and people in almost all industries and occupations will be affected. Some industries may be even reshaped, thus causing severe unemployment problems [4]. The implementation of AI reform in enterprises will undoubtedly exert different influences on employees from previous technological advances. The existing literature on the influences of AI development on employees focuses on four aspects. First, some studies have focused on the negative impacts of AI development on employees’ employment. For example, Chen and Xu speculated that in the next 20 years, 76.76% of China’s employed population will be replaced by AI [4]; Li et al. [5] and Xie et al. [6] found that enterprises adopting AI technology reduce the demand for low-skilled employees; and Huang speculated that with the development of AI technology, the income gap between high-skilled and low-skilled workers will increase, which will further intensify income inequality [7]. Second, some studies have focused on the positive impacts of AI development on employees’ employment. For example, Li et al. found that with the increasing application of AI, the demand for highly skilled labor increases [5]; He et al. found that the adoption of AI technology is positively correlated with employee income growth [3]; moreover, Mokyr et al. speculated that AI technology may create new products and services, leading new occupations to emerge [8]. Third, some studies have focused on the negative impacts of AI development on employees’ psychology and behavior. For example, Duan and Guo speculated that many employees may experience disappointment due to unemployment caused by AI [9]; Zhu et al. found that the application of AI trigger employees’ negative emotions [10]; Wang et al. found that employees are faced with high job insecurity due to large-scale utilization of AI technology [11]; Patel et al. found that the risk of job automation caused by AI technology harms employees’ health [12]; Brougham and Haar [13,14] and Li et al. [15] found that the likelihood of AI impacting employees’ career prospects was negatively related to organizational commitment and career satisfaction and positively related to turnover intentions, cynicism, and depression; Zhou et al. speculated that the technical attributes of algorithmic management that integrate big data and AI positively affect employees’ sense of unfairness in algorithms, which in turn induces job burnout [16]; and liu et al. speculated that online employment platforms strengthen the labor-process control of platform practitioners through algorithm technology, which makes platform practitioners’ work autonomy limited [17]. Fourth, other studies have explored the positive effects of AI development on employees’ psychology and behavior. For example, Zhu et al. found that the introduction of AI into the workplace can increase employees’ skill requirements for creativity and human–machine collaboration, which directly strengthens their thriving at work [18]; Zhou and Wang found that combining low-use-density and -intelligence robots enhances employees’ job performance by increasing their growth-need strength [19]; and Zhou et al. [16] and Goods et al. [20] speculated that algorithm management based on AI provides platform practitioners with the options of when, where, and how long to work, thus invigorating their job autonomy. A summary of the impacts of AI development on employees is shown in Table 1.

Our literature review indicates that, despite the negative impacts brought by the development and wide popularization of AI technology on employees, such as the considerable threats to employees’ employment, income, and autonomy [9,17], as a new type of intelligence, AI technology not only can improve the production efficiency of different industries and the skill level of employees but also create new industries and jobs [8,9,21]. In the process, those who have mastered the relevant knowledge and skills required in the age of AI are likely to benefit [22,23]. Therefore, in the context of AI transformation, individuals’ responsiveness to seize opportunities will provide motivation for their involvement in transforming their own personal professional knowledge and skills for individual professional advancement, resulting in a stronger sense of workplace well-being (WWB). However, few studies have explored the association between AI development and employees’ WWB. Based on the transactional theory of stress and resource conservation theory, we conceptualized the perception of AI opportunity and explored its association with employees’ WWB, as well as the mediating role of informal learning in the workplace (ILW) on the relationship between AI opportunity perception and employees’ WWB. In addition, we also examined the moderating effects of unemployment risk perception (URP) on the relationship between ILW and employees’ WWB, as well as the indirect relationship between AI opportunity perception and employees’ WWB via ILW. First, our research helps to further our understanding of the relationship between AI development and employees’ WWB. Second, it could help to formulate policies that guide employees to recognize AI technology and take measures to actively respond, thereby improving WWB and promoting the smooth application of AI technology in the workplace.

## 2. Theories Background and Hypotheses

### 2.1. Relationship between AI Opportunity Perception and Employees’ WWB

The transactional theory of stress explains (a) how individuals process stress and (b) the short- and long-term effects of stress. Stress is not derived from the situation itself—it is a subjective feeling that occurs when people feel that they lack the resources to cope with a situation that is important to them [24]. This theory holds that the interaction between individuals and stressful environments mainly involves two processes: cognitive appraisal and coping [25]. Cognitive appraisal primarily refers to the individual’s appraisal of the impact of external stressors on individual goals, values, and beliefs, including primary and secondary appraisal. The primary evaluation focuses on assessing whether the situation will affect the individual’s well-being. Secondary appraisal then focuses on whether the individual can control the outcome. The stressor is evaluated as a threat when the individual feels that there will be a bad outcome. When an individual believes that a stressor can cause them losses but also provides an opportunity to bring about gains, the stressor is assessed as a challenge [26]. Coping includes emotion- and problem-centered coping styles. The former includes escape, daydreaming, shirking responsibility, etc. The latter includes information gathering, seeking advice, summarizing experiences, and solving problems. An individual’s choice of coping style depends on how they evaluate the situation. Emotion-centered coping strategies are used more often when appraising the situation as a threat. When individuals believe that the situation can be changed and has an opportunity to bring about a gain, more problem-oriented coping strategies are used [26,27]. Coping often has a long-term impact on the individual. Generally speaking, problem-oriented coping will bring satisfactory results to the individual and improve their well-being [28]. Therefore, based on the transactional theory of stress, it can be inferred that when individuals believe there are opportunities in the situation and they can benefit from them, they tend to adopt problem-oriented coping styles, which have a positive impact on their well-being.

Because AI will replace some jobs, it will affect individuals’ jobs and income, which will cause considerable stress [9]. According to the transactional theory of stress, the impact of AI development on employees will depend on their cognitive appraisal and coping processes. AI has positive implications for workers, such as generating new employment opportunities [21,29] and increasing the income of some management and skilled positions [22,23]; however, its downsides include the replacement of jobs, leading to unemployment [30]. According to the transactional theory of stress, when individuals perceive that AI offers them opportunities, they will apply problem-oriented coping strategies, which will lessen their stress and improve their long-term well-being [28]. Penley, Tomaka, and Wiebe also found that when individuals adopt problem-oriented solving strategies to face stressors at work, they improve their physical and mental health [31] and, in doing so, reduce their level of work burnout [32]. Based on these studies, we propose the following hypothesis:

**H1.**  *After controlling for some variables, AI opportunity perception will be positively correlated with employees’ perceptions of WWB*.

### 2.2. Mediating Role of ILW

The transactional theory of stress holds that employees will adopt problem-oriented coping strategies when they believe that stressful situations present opportunities. Many studies have shown that learning is a crucial way to deal with AI transformation. For example, Frey et al. pointed out that the impact of AI technology on the labor market requires workers to reconsider their career choices, inspiring them to seize new employment opportunities through improving creative thinking and social skills [33]; additionally, Mao and Hu argue that it is necessary to take active measures to upgrade skills to seek new job opportunities for human resource management practitioners [29]. Learning is the primary method to build up knowledge reserves and skills. Learning at the workplace generally takes two forms: formal and informal learning. Formal learning typically refers to curricular behaviors and activities undertaken in a formally designated learning environment to acquire knowledge and skills, while informal learning behaviors are non-curricular behaviors and activities performed outside a designated learning environment to develop knowledge and skills. Informal learning emphasizes self-directed, intentional, and practice-based learning [34]. Formal learning often has many limitations, for example, many learning programs or courses may not meet the learning needs of employees [35]. On the other hand, informal learning emphasizes self-initiation, intrinsic motivation, personal control, and learning aimed at achieving the goals set by the learner [34]; therefore, informal learning is more important than formal learning to organizations and employees. In the context of AI transformation, informal learning is an effective way to transform the structure of knowledge and skills, and it can effectively help individuals seize the opportunities brought about by the development of AI. Therefore, when faced with the pressures placed by AI on workers, the higher the degree of opportunity employees perceive, the more problem-oriented coping strategies they will adopt, and the higher the likelihood of ILW.

WWB refers to employees’ perceptions and feelings about work satisfaction [36]. Previous studies have shown that informal learning in the workplace can improve individual job satisfaction [34], thereby enhancing employees’ WWB [37]. Self-determination theory states that humans have three psychological needs: competence, autonomy, and relatedness. Individuals will be prompted to establish a harmonious relationship with the environment when these needs are met, thereby enhancing their well-being [38,39]. In the work environment, competency refers to a person’s experience of a sense of efficacy at work; autonomy refers to a sense of control over the work environment; and relatedness refers to a person’s belief that one can build good interpersonal relationships at work [39]. Organizations implementing AI transformation require employees to acquire new knowledge and skills. ILW can help individuals to develop such new knowledge and skills, build their core technical knowledge and skills or expertise [34], and improve their individual work performance [40]. Zhu et al. hold that intelligent machines share employees’ repetitive and simple work, enabling employees to focus on learning and applying new skills, thereby building a sense of self-efficacy and competence [18]. ILW also contributes to the improvement of employee autonomy. First, after the introduction of AI technology or equipment in enterprises, the upgrading of job skill requirements prompts employees to generate high job enthusiasm and strive to be involved in various informal learning opportunities at the workplace to improve their skills, gradually adapt to changes, and gain greater control over their work environment [18], thus augmenting their sense of job autonomy. Second, learning to master new digital technologies, such as intelligent online office tools, can help employees work more efficiently given time and space limitations, strengthening their independence to solve work problems, thus invigorating their sense of job autonomy [41]. Previous studies have also substantiated that AI-based algorithm management can improve practitioners’ sense of job autonomy [16,20]. Informal learning that occurs in social interactions requires building good interpersonal relationships, and thus it may help meet relational needs [42]. Therefore, ILW can promote the satisfaction of three psychological needs and, in doing so, enhance employees’ WWB. Based on our above analysis, we found that AI opportunity perception was positively related to ILW, and ILW was positively related to employees’ WWB. Therefore we propose the following hypothesis:

**H2.**  *ILW plays a mediating role in the relationship between AI opportunity perception and employees’ WWB*.

### 2.3. Moderating Effect of Unemployment Risk Perception

Resource conservation theory argues that worries about job instability and persistence activate the resource consumption process, leading to emotional exhaustion in individuals [43,44]. When emotional exhaustion occurs, individuals tend to adopt defensive strategies (i.e., withholding resource investment by not exerting effort) to prevent further consumption of resources [45], which, in turn, can adversely affect the individual.

The introduction of AI technology has two results. On the one hand, it will bring development opportunities to employees [21,29]; however, employees will also face the threat of unemployment [46]. If URP cannot be effectively controlled, employees will be emotionally exhausted and adopt defensive strategies to prevent the threat of losing resources. In such a situation, URP will reduce the likelihood of employees adopting a problem-oriented coping style, such as ILW, and thus mitigate employees’ WWB. Therefore, URP is likely to attenuate the impact of AI opportunity perception on ILW and the indirect impact on WWB through ILW. Based on the above analysis, this paper proposes the following hypotheses:

**H3.**  *URP negatively moderates the relationship between AI opportunity perception and ILW*.

**H4.**  *URP moderates the mediating relationship between AI opportunity perception and employees’ WWB via ILW*.

**Figure 1:**
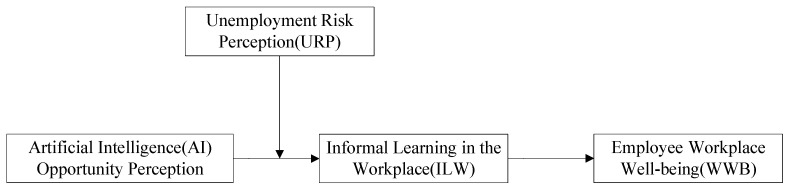
Theoretical model.

Accordingly, we propose the following theoretical model (Figure 1).

## 3. Methods

### 3.1. Procedure and Sample

**Figure 2:**
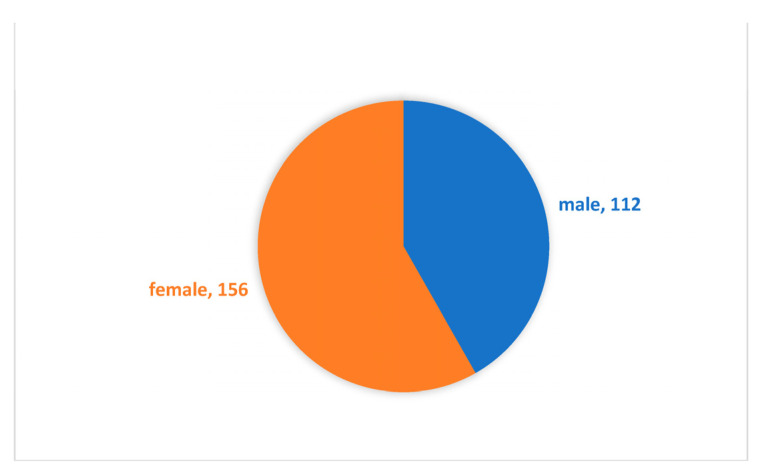
Distribution of gender (numbers in the figure represent an actual number of test subjects in each group).

**Figure 3:**
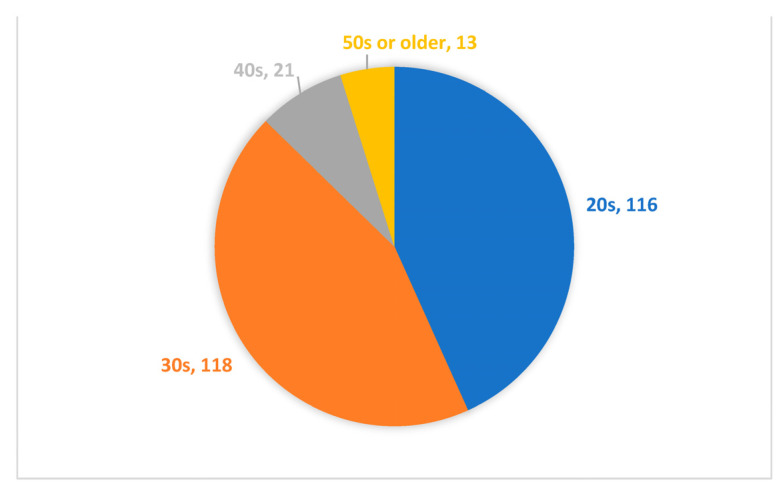
Distribution of age (numbers in figure represent an actual number of test subjects in each group).

**Figure 4:**
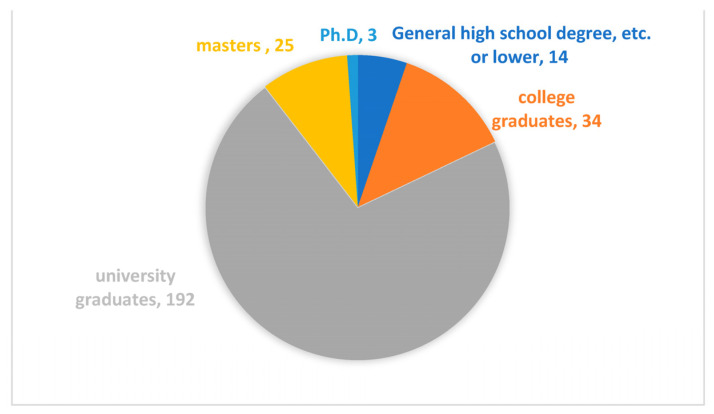
Distribution of educational qualifications (numbers in figure represent an actual number of test subjects in each group).

**Figure 5:**
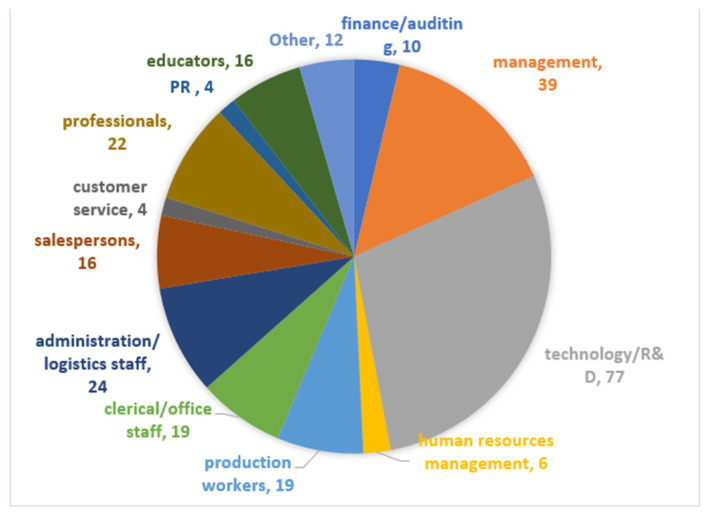
Distribution of occupation (numbers in figure represent an actual number of test subjects in each group).

We used the professional data-collection platform Credamo (https://www.credamo.com/, accessed on 23 December 2022) to administer the questionnaires to corporate employees. As a professional data platform providing nationwide large-scale data-collection services, Credamo has been widely recognized by Chinese scholars. Research results garnered through this platform have also been recognized by top international journals in the fields of management and psychology. We sent questionnaires to 300 participants. After, we excluded incomplete questionnaires, as well as questionnaires with contradictory answers. Finally, we used 268 completed questionnaires (response rate 89.33 %) in our research. Figure 2 illustrates the distribution of gender. Respondents were 41.8% male and 58.2% female. Figure 3 illustrates the distribution of age. The 30s were the largest group with 44%, followed by 43.3% in their 20s, 7.8% in their 40s, and 4.9% in their 50s or older. Figure 4 illustrates the distribution of educational qualifications. The group of university graduates was the highest with 71.6%, followed by 12.7% for college graduates, 5.3% for general high school/secondary school/technical school/vocational high school degrees (abbreviated to general high school degree, etc., in Figure 4) or lower, 9.3% for Master’s degree, and 1.1% for Ph.D. Figure 5 illustrates the distribution of occupation. Respondents came from a variety of occupations, including finance/auditing, management, technology/R&D, human resources management, production workers, clerical/office staff, administration/logistics staff, salespersons, customer service, professionals (such as accountants, lawyers, architects, healthcare workers, journalists), PR, educators, etc.

### 3.2. Measures

Workplace well-being (WWB): We used the scale developed by Zheng et al. [36] with six items. We asked the participants to choose the scale’s assessment of workplace well-being. We measured items on a 5-point Likert scale ranging from 1 (strongly disagree) to 5 (strongly agree). A sample item is: “I am satisfied with my work responsibilities” (see Appendix A). Cronbach’s alpha was 0.702.

Informal learning in the workplace (ILW): We measured ILW with nine items from the scale developed by Noe, Tews, and Marand [47]. The scale encouraged the participants to consider how often they participated in informal learning activities during a typical working week in the past three months. We measured items on a 5-point Likert scale ranging from 1 (never) to 5 (all the time). A sample item is: “ Reflecting about how to improve my performance” (see Appendix A). Cronbach’s alpha was 0.732.

AI opportunity perception: We measured AI opportunity perception with the scale developed by Highhouse and Payam [48] with five items, which we revised according to the research background of AI technology development. For example, we changed the item “opportunity” from the original scale to “It is an opportunity for me that enterprises apply artificial intelligence” (see Appendix A). We measured items on a 5-point Likert scale ranging from 1 (strongly disagree) to 5 (strongly agree). Cronbach’s alpha was 0.702.

Unemployment risk perception (URP): To examine the extent to which employees perceived their unemployment risk, we asked them to evaluate their agreement with a set of statements. We adapted the three statements from previous research [49]. For example, we changed the item “How likely do you think you are to get [bird flu]?” from the original scale to “I am likely to lose my job because of the development of artificial intelligence” (see Appendix A). We measured items on a 5-point Likert scale ranging from 1 (strongly disagree) to 5 (strongly agree). Cronbach’s alpha was 0.816.

Control variables: We controlled for essential demographic variables related to WWB, such as gender, education level, and age [50].

### 3.3. Analytical Strategy

We used SPSS version 25.0, MPLUS version 8.3, and PROCESS version 3.4 macro program to analyze the data. First, to test the discriminant validity of the four variables (AI opportunity perception, ILW, URP, WWB), we performed confirmatory factor analysis (CFA) using MPLUS version 8.3. Second, we used descriptive statistics, correlation, and regression analyses to examine the relationship between variables with SPSS version 25.0. Finally, we used PROCESS version 3.4 macro program to test the mediating, moderating, and moderated mediating effects.

## 4. Results

### 4.1. Confirmatory Factor Analysis

To test the discriminant validity among the four variables, namely AI opportunity perception, ILW, URP, and WWB, we performed confirmatory factor analysis using MPLUS version 8.3. Our results showed (see Table 2) that the four-factor measurement model fit the data well (χ^2^ = 263.24, df = 164, CFI = 0.93, TLI = 0.92, RMSEA = 0.05). Results of model comparisons showed that the hypothesized four-factor measurement model best fit the data (△χ^2^ [3 < Δdf < 6] ranged from 35.64 to 322.77, *p* < 0.01). The four key variables of this study had good discriminant validity.

### 4.2. Common Method Bias

To avoid common method bias affecting the research conclusions, we used Harman’s single-factor test for common methodological bias. The results showed that the variance explained by the first factor was 26.42%, which did not exceed 40%, indicating there was no serious common method bias. 

### 4.3. Descriptive Statistics and Correlation Analysis

The variables’ means, standard deviations, and correlations are shown in Table 3. We used the Bonferroni *p* = 0.001 to evaluate the significance of each correlation to keep the overall type-1 error rate approximately equal at a 0.05 level for 42 tests. As the table shows, AI opportunity perception was significantly positively correlated with ILW (r = 0.45, *p* = 0.000) and significantly correlated with WWB (r = 0.59, *p* = 0.000); ILW was significantly positively correlated with WWB (r = 0.62, *p* = 0.000); and URP was significantly negatively correlated with AI opportunity perception (r = −0.63, *p* = 0.000) and WWB (r = −0.40, *p* = 0.000).

### 4.4. Hypotheses Testing

To test Hypothesis 1, we took gender, education level, and age as the control variables, AI opportunity perception as an independent variable, and WWB as a dependent variable for regression analysis. Model 1 in Table 4 shows that after controlling for some variables, AI opportunity perception is positively related to WWB (β = 0.61, *p* = 0.000). Hypothesis H1 was thus verified.

We used the method proposed by Baron and Kenny to test the mediating effect of ILW [51]. Table 4 shows the results. Model 2 shows that after controlling for gender, education level, and age, AI opportunity perception was positively related to ILW (β = 0.45, *p* = 0.000). Then, Model 3 shows that when AI opportunity perception was controlled, ILW was positively related to employees’ WWB (β = 0.44, *p* = 0.000), and the relationship between AI opportunity perception and employees’ WWB was significant (β = 0.41, *p* = 0.000). These results show that ILW played a mediating role between AI opportunity perception and employees’ WWB, and it had a partial mediating effect. Hypothesis H2 was thus verified.

To further ensure the reliability of our empirical results, we used SPSS PROCESS version 3.4 macro programto again verify the mediating role of ILW. Our analysis results showed that the indirect effect between AI opportunity perception and employees’ WWB via ILW was 0.17, 99% CI = 0.10–0.28, which verified Hypothesis H2 again. 

To test the moderating effect of URP, using a general test of the moderating effect, we standardized AI opportunity perception and URP through converting the raw scores into Z scores. Then, we conducted a regression analysis. Table 4 shows the results. Model 5 shows that after controlling for gender, education level, and age, the interaction items of AI opportunity perception and URP were significantly related to the ILW (β = −0.35, *p* = 0.000). As such, URP played a moderating role in the relationship between AI opportunity perception and ILW, which verified Hypothesis H3.

**Figure 6:**
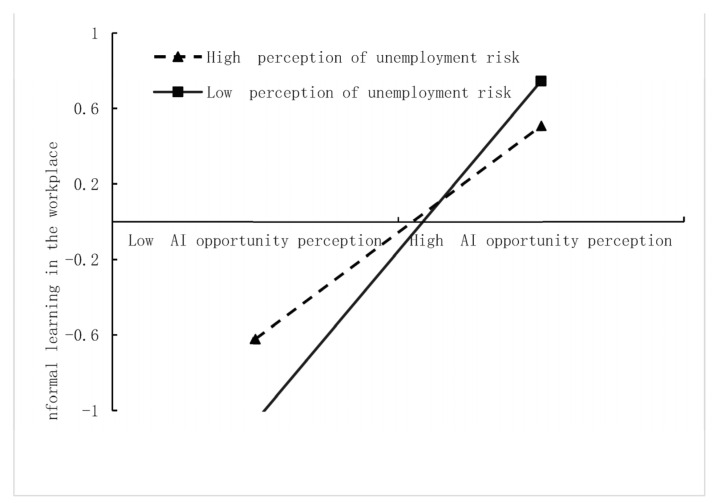
Interaction effect of URP on the relationship between AI opportunity perception and ILW.

To further clarify the direction and size of the moderating effect, we standardized the AI opportunity perception, URP, and ILW through converting the raw scores into Z scores. The simple slope test results showed that when the URP value was lower, the relationship between AI opportunity perception and ILW was positive and significant and the effect was greater (β = 0.90, *p* = 0.000); when the URP value was higher, the relationship between AI opportunity perception and ILW was also positive and significant but the effect was smaller (β = 0.57, *p* = 0.000). The specific visualization results are shown in Figure 6. When the unemployment risk perception is high, AI opportunity perception is positively related to ILW. The same pattern occurs for low URP but with a steeper slope. This shows that URP negatively moderated the relationship between AI opportunity perception and ILW, which again supported Hypothesis H3.

Hypothesis H4 proposed that URP moderates the mediating role of ILW between AI opportunity perception and WWB. To test this hypothesis, we used SPSS PROCESS Version 3.4 macro program to test the moderated mediation. Our results showed that when the URP value was lower, the indirect effect between AI opportunity perception and WWB via ILW was 0.33, with a 99% confidence interval of 0.18–0.52; when the URP value was higher, the indirect effect between AI opportunity perception and WWB via ILW was 0.21, with a 99% confidence interval of 0.11–0.33. The difference in the indirect effect between AI opportunity perception and WWB via ILW under the conditions of higher and lower URP values was −0.12, and the 99% confidence interval ranged from −0.23 to −0.05. Our findings suggest that the indirect effect of ILW between AI opportunity perception and WWB was significantly different when employees had different levels of URP. The lower the UPR value was, the greater the indirect effect of ILW between AI opportunity perception and WWB via ILW. Therefore, URP negatively moderated the mediating role of ILW between AI opportunity perception and WWB. As such, hypothesis H4 was verified.

## 5. Discussion

Although the concept of “AI” was put forward in 1956, it was not until the 1990s that this technology rapidly developed and was extensively applied in enterprise management and production [1]. As AI technology will replace many jobs while improving production and management efficiency, leading to the threat of unemployment for employees in enterprises, researchers have begun to focus on the impact of AI technology on employees. From the opportunity perception of AI perspective, we discussed the relationships between AI development and employees’ WWB. Specifically, we tested four hypotheses and found that our results supported them. 

Our results revealed that AI opportunity perception had a significant positive correlation with employees’ WWB. To our knowledge, there has been no research on the relationship between AI development and employees’ WWB. However, Zhu et al. argued that pursuing improvements to job skills relevant to AI is conducive for employees to eliminate repetitive and complicated work, resulting in a pleasant psychological experience, such as feeling competent and thriving at work [18]. Additionally, Brougham and Haar verified that AI threat perception is negatively associated with career satisfaction [13], which means the inverse is true for AI opportunity perception. These research conclusions are consistent with our findings.

ILW played a mediating role in the relationship between AI opportunity perception and employees’ WWB. Previous research showed that employees need to learn and improve their knowledge and skill structures to adapt to the new challenging working environments created by the AI revolution [29]. These results suggested that employees’ strong consciousness of career growth opportunities brought by AI promoted their ILW. Previous studies found that ILW is positively correlated with employees’ job satisfaction [34], while job satisfaction is positively correlated with employees’ WWB [52]. These results are also consistent with our findings. 

URP negatively moderated the mediating role of ILW between AI opportunity perception and WWB, which suggests that URP can weaken the relationship between AI opportunity perception and employees’ WWB. Previous studies suggested that unemployment and job insecurity can both exert negative impacts on well-being at work [53,54]. Our study showed that URP was not only directly negatively related to employees’ WWB but also impaired the positive relationship between other factors and employees’ WWB. However, it should be noted that in terms of the educational distribution of the samples, those with a university education or higher accounted for a relatively high proportion of respondents (82.9%). Some studies have shown that the higher the education level is, the lower the unemployment risk perceived by individuals will be [46], which means that the URP of the samples studied in this paper is mainly distributed at a lower level. Nonetheless, our conclusion similarly suggests that URP can weaken the relationship between the perception of AI opportunities and employees’ WWB, which shows that although the URP of the main highly educated group is lower, it is also necessary to pay attention to the weakening effect of URP on the relationship between AI opportunity perception and employees’ WWB.

### 5.1. Theoretical Implications

Based on the transactional theory of stress, we explored the relationship between AI opportunity perception and employees’ WWB and verified the mediating role of ILW and the moderating role of URP, thereby extending the related research on employees’ WWB. The theoretical implications of our study are mainly reflected in the following four aspects: 

First, we extended the knowledge of the relationship between AI development and employees’ psychology and behavior. With the growing popularity of AI technology among enterprises, many scholars have begun to explore the impact of AI on employees, including negative emotions [11], health status [12], organizational identity, career satisfaction, turnover intention, and cynicism [13,14,15]. However, few studies have explored the possibility of a relationship between AI and employees’ WWB. From the perspective of AI opportunity perception, we discussed the relationship between AI and employees’ WWB, and contributed towards advancing the related research on the impact of AI development.

Second, we furthered the understanding of employee coping behaviors in AI stress situations. Previously, the research on employees’ coping behaviors in stressful situations mainly focused on planned problem-solving, information collection, and positive stress reassessment [55]. We suggested that AI opportunity perception is positively related to ILW. As such, we extended the understanding of how employees respond to stressful situations under AI transformation scenarios.

Third, we furthered the understanding of the consequences of ILW. In the past, the impact of ILW was understood mainly in relation to job performance and the individual’s acquisition of knowledge and skills [37]. We found that ILW is positively related to employees’ WWB.

Fourth, our study extended the knowledge of the conditions that support employees’ WWB in the context of AI transformation. AI opportunity perception was positively related to employees’ ILW and WWB, and employees’ URP moderated this relationship. This helps clarify the constraints on the positive relationship between AI opportunity perception and employees’ WWB and provides a theoretical basis for actively guiding employees to cope with the development of AI.

### 5.2. Management Implications

Our research results provide several ideas for enterprises to promote employees’ active response and improve employees’ WWB in the context of AI development.

First, enterprises should actively guide employees to understand the opportunities brought by AI transformation for their career development. Our results showed that the higher the degree of employees’ AI opportunity perception, the better employees’ WWB. This suggests that companies should take measures to help employees evaluate AI transformation as an “opportunity” conducive to improving their WWB. 

Second, enterprises should create an informal learning atmosphere in the workplace for employees. Our findings suggested that AI opportunity perception is positively related to employees’ WWB, which is mediated by ILW. ILW not only requires employees to generate active learning motivation but also requires enterprises to create a good atmosphere to strengthen and guide employees to actively adopt informal learning methods in the workplace to deal with AI transformation. Generally speaking, informal learning includes daily behavioral reflection, self-study, seeking feedback, knowledge-sharing activities, etc. [56]. To create an informal learning atmosphere to guide and motivate employees to learn informally, employee study groups could be established, encouraging workers to summarize and share work experiences. Furthermore, a corporate library could be opened to guide employees to update their knowledge through self-study. Additionally, a relaxed and open organizational atmosphere could be established to encourage employees to actively seek feedback from colleagues and leaders.

Third, enterprises should reduce employees’ URP. Our findings suggested that URP values negatively moderate the relationship between AI opportunity perception and ILW, along with the indirect effect of ILW between AI opportunity perception and WWB. Therefore, while guiding employees to face the opportunities brought by AI technology, enterprises also need to minimize employees’ URP. To achieve this goal, first, enterprises should actively train employees on AI to enhance their confidence in coping with AI; second, enterprises should take measures to reduce employees’ uncertainty perception brought about by AI changes. To do so, enterprises can, for instance, establish new career development paths for employees to prevent them from losing their jobs due to the development of AI.

### 5.3. Limitations and Future Research Directions

The limitations of this study mainly include the following:

First, our study employed self-report measures. Despite our efforts to minimize the common method bias, future research can increase reliability through a third-party evaluation.

Second, in terms of the educational level of the samples, the proportion of people with general high school/secondary school/technical school/vocational high school degrees or lower is relatively low. This may have limited the generalizability of the findings for people with broader employees. In the future, consideration will be given to increasing the number of employees with general high school/secondary school/technical school/vocational high school degrees or lower through more investigation channels to make the participants’ educational background distribution more balanced.

Third, we mainly explored the mediating role of ILW in the relationship between AI opportunity perception and employees’ WWB; however, there may be other mediating mechanisms. In the future, researchers should consider exploring the mediating roles of job crafting [39], cognitive reappraisal [55], and other variables.

Finally, we explored the moderating role of URP in the relationship between AI opportunity perception and employees’ WWB. Based on the transactional theory of stress, self-confidence, core self-evaluations, and self-esteem may all be related to employees’ assessment of stressful events [55]. In addition, AI development may not only cause employees’ URP but may also induce the risk of decreasing employee autonomy [17], thus reducing the relationship between AI opportunity perception and employees’ WWB. Researchers should continue to explore the moderating roles of these variables in the relationship between AI opportunity perception and employees’ WWB in future studies.

## 6. Conclusions

Our study discussed the relationship between AI development and employees’ WWB from the perspective of AI opportunity perception. Based on the transactional theory of stress and resource conservation theory, we constructed a moderated mediation model, which includes AI opportunity perception, ILW, URP, and WWB. Our results from a survey of 268 employees confirmed our proposed hypotheses. Furthermore, our study extended earlier work on the relationship between AI development and workplace outcomes, and our results supported the association between AI opportunity perception and employees’ WWB. We hope that the results of our study can help researchers to replicate and extend our research. In addition, we also hope our research can help the formulation of policies that guide employees to recognize AI technology and adopt effective measures to cope with rapidly changing technologies, thereby improving their WWB.

## Figures and Tables

**Table 1:** Summary of impacts of the development of artificial intelligence on employees.

Impacts of the Development of AI on Employees	Empirical Findings	Speculation
Negative impacts of AI development on employees’ employment	Reduces the demand for low-skilled employees [5,6];increases income inequality between high-skilled and low-skilled workers [7]	Replaces workers [4]
Positive impacts of AI development on employees’ employment	Increases the demand for highly skilled labor [5]; increases the income of employees [3]	Spawns new occupations [8];
Negative impacts of AI development on employees’ psychology and behavior	Triggers employees’ negative emotions [10]; increases employee job insecurity [11]; harms employees’ health [12]; reduces employees’ organizational identity and career satisfaction, and increases their turnover intention, cynicism, and depression [13,14,15]	Increases employee disappointment [9]; induces job burnout [16]; strengthens labor-process control [17]
Positive impacts of AI development on employees’ psychology and behavior	Employees thrive at work [18]; which enhances employees’ job performance [19]	Improves job autonomy [16,20]

**Table 2:** Confirmatory factor analysis.

Model	Factor	χ^2^	df	CFI	TLI	RMSEA
Four-factor model	AI opportunity perception, ILW, URP, WWB	263.24	164	0.93	0.92	0.05
Three-factor model	AI opportunity perception + URP, ILW, WWB	343.21	167	0.88	0.86	0.06
Three-factor model	AI opportunity perception + ILW, URP, WWB	420.69	167	0.82	0.80	0.08
Three-factor model	AI opportunity perception, ILW + WWB, URP	298.88	167	0.91	0.89	0.05
Two-factor model	AI opportunity perception + URP, WWB + ILW	377.86	169	0.85	0.83	0.07
One-factor model	AI opportunity perception + ILW + URP + WWB	586.01	170	0.71	0.67	0.10

Note: *N* = 268; AI refers to artificial intelligence; ILW refers to informal learning in workplace; URP refers to unemployment risk perception; WWB refers to workplace well-being.

**Table 3:** Means, standard deviations, and correlations.

Variables	Mean	SD	1	2	3	4	5	6
1. Gender	0.42	0.50						
2. Education level	4.88	0.69	0.08					
3. Age	31.90	7.27	0.01	−0.36 *				
4. AI opportunity perception	4.21	0.51	0.10	0.14	−0.04			
5. ILW	4.22	0.46	0.16	−0.03	−0.00	0.45 *		
6. URP	1.88	0.78	−0.06	−0.13	0.01	−0.63 *	−0.19	
7. WWB	4.25	0.44	0.07	−0.02	0.05	0.59 *	0.62 *	−0.40 *

Note: *N* = 268, * *p* < 0.001. SD = standard deviation. Gender is coded 1 = male and 0 = female; education level is coded 1 = primary school or below, 2 = junior high school, 3 = general high school/secondary school/technical school/vocational high school, 4 = college graduates, 5 = university graduates, 6 = Master’s degree, 7 = Ph.D.

**Table 4:** Hierarchical regression analysis results of variables.

	Model 1: WWB	Model 2: ILW	Model 3: WWB	Model 4: ILW	Model 5: ILW
Gender	0.01 (0.858)	0.13 (0.023)	−0.046 (0.292)	0.12 (0.024)	0.08 (0.156)
Education level	−0.09 (0.106)	−0.11 (0.056)	−0.04 (0.437)	−0.10 (0.075)	−0.13 (0.026)
Age	0.036 (0.491)	−0.03 (0.611)	0.05 (0.285)	−0.02 (0.675)	−0.02 (0.665)
AI opportunity perception	0.61 (0.000)	0.45 (0.000)	0.41 (0.000)	0.54 (0.000)	0.73 (0.000)
ILW			0.44 (0.000)		
URP				0.14 (0.041)	0.05 (0.470)
URP × AI opportunity perception					−0.35 (0.000)
R^2^	0.36 (0.000)	0.23 (0.000)	0.52 (0.000)	0.24 (0.000)	0.22 (0.000)
F	37.54	19.494	55.54	16.628	18.246

Note: *p* values are in parenthesis.

## Data Availability

The data presented in this study are available on request from the corresponding author.

## Appendix A

**Table A1:** Measurement items of key variables.

Variables	Items		Source
Artificial Intelligence (AI) Opportunity Perception	1	The adoption of artificial intelligence by enterprises is beneficial to me;	Highhouse and Payam (1996) [48]
	2	The influence of enterprises applying artificial intelligence on me can be controlled;	
	3	The application of artificial intelligence by enterprises can increase the likelihood of my personal successful career development;	
	4	It is an opportunity for me that enterprises apply artificial intelligence;	
	5	It is possible for me to gain more than lose when enterprises apply artificial intelligence.	
Informal Learning in the Workplace (ILW)	1	Reflecting about how to improve my performance;	Noe, Tews and Marand (2013) [47]
	2	Experimenting with new ways of performing my work;	
	3	Using trial and error strategies to learn and perform better;	
	4	Interacting with a mentor;	
	5	Interacting with my supervisor;	
	6	Interacting with my peers;	
	7	Reading professional magazines and vendor publications;	
	8	Searching the Internet for job-relevant information;	
	9	Reading management books.	
Workplace Well-being (WWB)	1	I am satisfied with my work responsibilities;	Zheng et al. (2015) [36]
	2	I feel basically satisfied with my work achievements in my current job;	
	3	I find real enjoyment in my work;	
	4	I can always find ways to enrich my work;	
	5	Work is a meaningful experience for me;	
	6	In general, I feel fairly satisfied with my present job.	
Unemployment Risk Perception (URP)	1	I am likely to lose my job because of the development of artificial intelligence;	Hovick et al. (2011) [49]
	2	I am worried about losing my job because of the development of artificial intelligence;	
	3	Compared with other people in the same profession, the development of artificial intelligence is more likely to cause me to lose my job.	
